# Innate and Adaptive Immunity in Aging and Longevity: The Foundation of Resilience

**DOI:** 10.14336/AD.2020.0603

**Published:** 2020-12-01

**Authors:** Alexey Moskalev, Ilia Stambler, Calogero Caruso

**Affiliations:** ^1^Institute of Biology of FRC of Komi Scientific Center of Ural Branch of Russian Academy of Sciences, Syktyvkar, 167982, Russia.; ^2^Vetek (Seniority), The Movement for Longevity and Quality of Life, Israel.; ^3^Laboratory of Immunopathology and Immunosenescence, Department of Biomedicine, Neurosciences and Advanced Diagnostics, University of Palermo, Palermo, Italy

**Keywords:** innate immunity, adaptive immunity, aging, longevity, resilience

## Abstract

The interrelation of the processes of immunity and senescence now receives an unprecedented emphasis during the COVID-19 pandemic, which brings to the fore the critical need to combat immunosenescence and improve the immune function and resilience of older persons. Here we review the historical origins and the current state of the science of innate and adaptive immunity in aging and longevity. From the modern point of view, innate and adaptive immunity are not only affected by aging but also are important parts of its underlying mechanisms. Excessive levels or activity of antimicrobial peptides, C-reactive protein, complement system, TLR/NF-κB, cGAS/STING/IFN 1,3 and AGEs/RAGE pathways, myeloid cells and NLRP3 inflammasome, declined levels of NK cells in innate immunity, thymus involution and decreased amount of naive T-cells in adaptive immunity, are biomarkers of aging and predisposition factors for cellular senescence and aging-related pathologies. Long-living species, human centenarians, and women are characterized by less inflamm-aging and decelerated immunosenescence. Despite recent progress in understanding, the harmonious theory of immunosenescence is still developing. Geroprotectors targeting these mechanisms are just emerging and are comprehensively discussed in this article.

The interrelation of the processes of immunity and senescence now receives an unprecedented emphasis during the COVID-19 pandemic, which has stressed the critical need to combat immunosenescence and improve the immune function and resilience of older persons. At this time, it is appropriate to review the current state of the science of innate and adaptive immunity in aging and longevity, as well as the historical origins of this field of study, to further promote the research in this area. That is the subject of the present work.

Historically, the field originated at the turn of the 20^th^ century with the work of Elie Metchnikoff whose 175^th^ anniversary we celebrated on May 15, 2020 (May 15, 1845 - July 15, 1916). Metchnikoff is well recognized as a pioneering immunologist and microbiologist, a vice-director of the Pasteur Institute in Paris, and the Nobel Laureate in Physiology or Medicine of 1908 for the discovery of phagocytosis (a major contribution to the cellular theory of immunity). Yet, he may also be well credited as “the father” of gerontology - the disciplinary term he coined. Both the terms “gerontology” (“the study of aging”) and “thanatology” (“the study of death”) were coined by him in the “Etudes On the Nature of Man” published in 1903, which may mark the beginning of these scientific fields. Moreover, Metchnikoff can also be credited for the establishment of the interdisciplinary connection between these fields, in particular between aging research and immunology. Metchnikoff was the author of arguably the first systematic scientific theory of aging, interrelating the processes of immunity and senescence (www.longevityhistory.com/) [1]. In Metchnikoff’s own words: “We saw that, during aging, there occurs a struggle between noble elements (parenchymal tissues, e.g. the tissues of the muscle, kidney, lung, and brain) and phagocytes (“low/primitive elements”), and that the vitality of the former is, for the most part, diminished, whereas the latter, on the contrary, show increased activity. Therefore, it would seem that the means to use in the struggle against pathological aging should be, on the one hand, the strengthening of the most valuable elements of the organism, and on the other, the attenuation of the aggressive onslaught of the phagocytes. I must point out to the reader from the beginning that this problem is not yet solved, but its solution does not involve anything impossible. It is a scientific question, like many others.” (Elie Metchnikoff, Etudy o Prirode Cheloveka (Etudes on the Nature of Man), The USSR Academy of Sciences Press, Moscow, 1961 (1903), Ch. X. “Vvedenie v nauchnoe izuchenie starosti” (An introduction to the scientific study of aging), pp. 201-202.)

Following a century of study, at the present time, natural immunity is understood to consist of three interrelated parts: physiological barriers, innate immunity and adaptive immunity. All of these are affected by aging [2]. Immunosenescence results in increased susceptibility and severity of infectious diseases and non-communicable age-associated diseases, among them cancer, cardio-vascular disease, and autoimmunity [3].

The molecular mechanisms of the induction of inflammation and cellular senescence intersect through activation of the TLR/NF-κB, cGAS/STING/IFN 1,3, AGEs/RAGE molecular signaling pathways and the assembly of the NLRP3 inflammasome. Chronic sterile inflammation with aging was termed by Claudio Franceschi “inflamm-aging” [4]. The hyperactivation of the innate immunity response predominantly reduces the lifespan.

Antimicrobial peptides are involved in chemotaxis and activation of innate and adaptive immunity cells in different animals from invertebrates to humans [5, 6]. On the model of *Drosophila*, it is shown that hyperactivation of different antimicrobial peptide genes significantly reduces the lifespan [7].

Pathogen-associated molecular patterns of microorganisms are recognized by the innate immune system through inherited pattern-recognition receptors [8], including C-reactive protein [9], Toll-like-receptors [10] and some cytoplasmic receptors, including cGas/STING [11].

**Figure 1.**
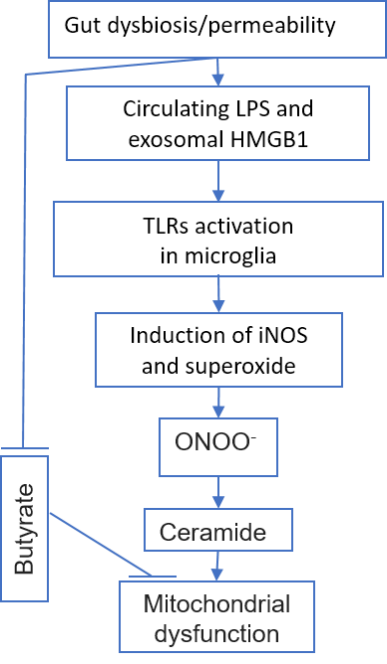
Gut dysbiosis/permeability with aging can induce TLR in microglia and exerts mitochondrial dysfunction.

C-reactive protein (CRP) is a soluble pathogen pattern recognition receptor. It binds to 1,6-Bis(phosphocholine) of cell membranes of damaged cells or bacteria to induce complement or immune cell activation [9]. CRP hyperactivity is a biomarker of aging and is connected with inflammation and fibrosis [12].

The complement system includes more than 50 proteins in the plasma and cell membrane that act in response to activation of pattern recognition receptors, including CRP, killing microbes, sending danger signals, and accelerating apoptosis of damaged cells. There was established the participation of the complement system in the pathogenesis of aging-dependent diseases and their complications, including age-related macular degeneration [13] and type 2 diabetes [14].

Toll-like receptors (TLR) cell surface receptors can recognize pathogen patterns from viruses, bacteria, or fungi to induce NF-kB proinflammatory signaling. TLR inhibition is a potential target to alleviate neuroinflammation [15]. TLR4 is of particular interest in connection with aging, since it can be activated by cytotoxic oxysterols (7-ketocholesterol), which are formed in tissues during inflammation or come from long-stored food [16]. Its activation leads to mitochondrial dysfunction and an inflammatory reaction [17], including in brain microglia [18] (Fig. 1). TLR4 is inhibited by substances from cocoa [16]. Some polyphenols can suppress overexpression of inflammatory mediators through TLR4/NF-κB/STAT signaling intervention [19].

**Figure 2.**
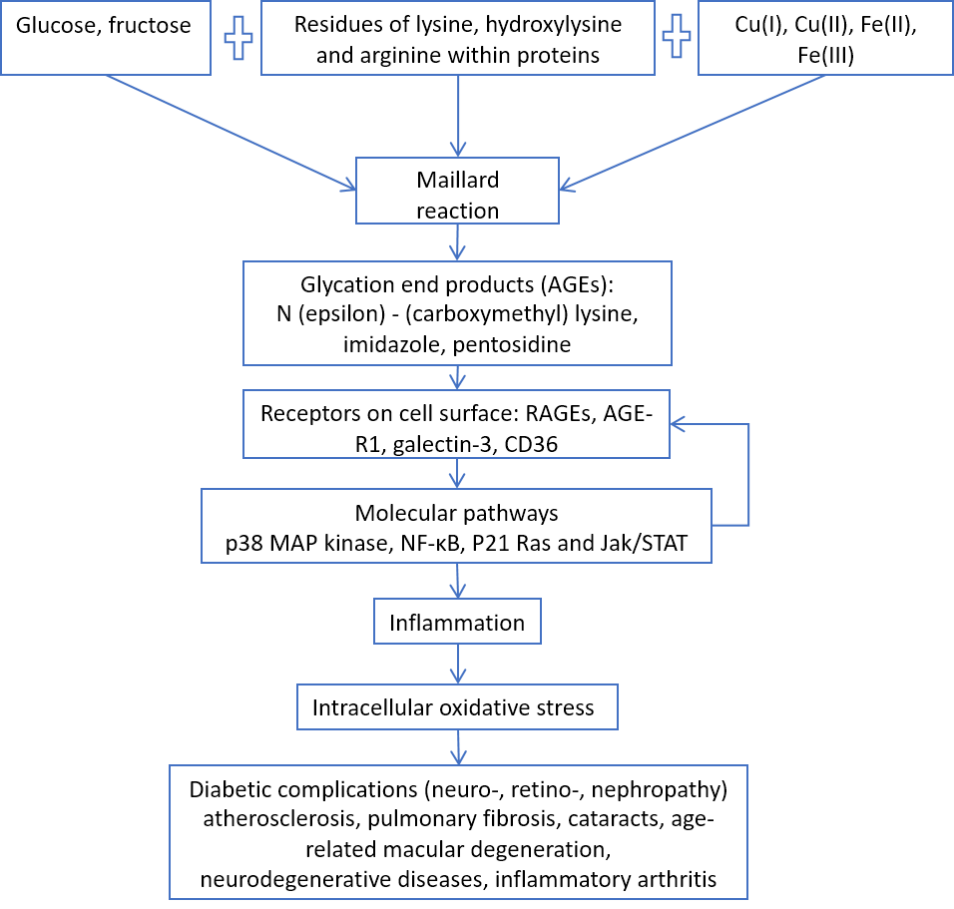
AGEs/RAGE pathway and age-related diseases.

cGas/STING pathway is the main intracellular sensor of viral invasion, including SARS-Cov-2, to induce Interferon 1 and 3 productions [20]. The STING pathway is also hyperactivated with aging by internal reasons, including retrotransposons, chromatin and mtDNA fragments in the cytosol with a consequent interferon induction, cellular senescence and apoptosis [21-24]. This could be a hypothetical reason for the greater severity of Covid-19 in elderly people.

Some simple sugars (glucose, fructose) in the presence of transition metal ions (iron and copper) react chemically with amino acid residues (lysine, arginine) in proteins, such as collagen and elastin, causing the formation of glycation end products that not only increase the extracellular matrix stiffness, but also induce chronic inflammation through their RAGE receptors on the surface of cells (Fig. 2), such as vascular endothelium [25].

Cellular senescence itself can induce inflammation by secreting pro-inflammatory cytokines, the so-called Senescence Associated Secretory Phenotype (SASP) [26] (Fig. 3).

According to Baker's study, elimination of senescent cells prolongs the lifespan and healthspan of mice [27, 28].

**Figure 3.**
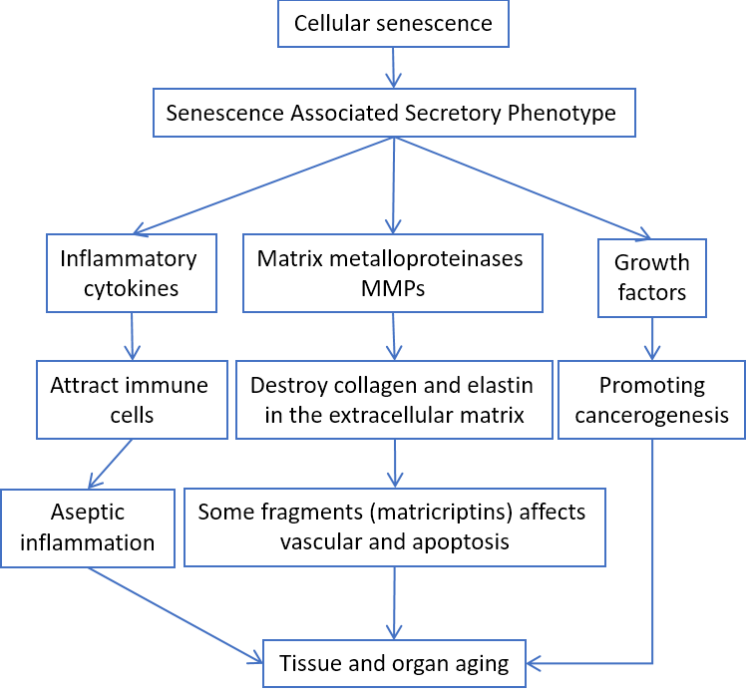
SASP involvement in aging-related pathologies.

Adiposity is another source of inflamm-aging. Adipocyte hypertrophy leads to the secretion of proinflammatory leptin, lipocalin-2, progranulin, and chemoattractants for T- and B-cells [29-33] (Fig. 4).

In addition, there are many other ways of induction of the main proinflammatory transcription factor NF-kB: overeating, obesity [34], dysbiosis [35], psychological and chronic stress [36], vitamin D deficiency [37], circadian rhythm disturbance [38], aldosterone [39], angiotensin II [40], mitochondrial N-formyl peptides [41], oxidized mitochondrial DNA [42] (Fig. 5).

In our experiments on the *Drosophila* model, we inhibited the different underlying pathways of NF-kB. In most cases, this led to an increase in lifespan [43-45].

It is worth noting that many substances of natural origin contained in food can inhibit NF-kB [46].

Aging is accompanied by gut microbiota alteration, like decreased overall diversity and an increased abundance of proinflammatory species, that can be a part of systemic inflammation and many aging-related diseases [47, 48].

Internal virome aging-related changes can affect different age-dependent diseases, including immune-senescence [49] and atherosclerosis [50] by cytomegalovirus, cancerogenesis by papillomaviruses [51] and Alzheimer’s by simple herpes [52-55].

**Figure 4.**
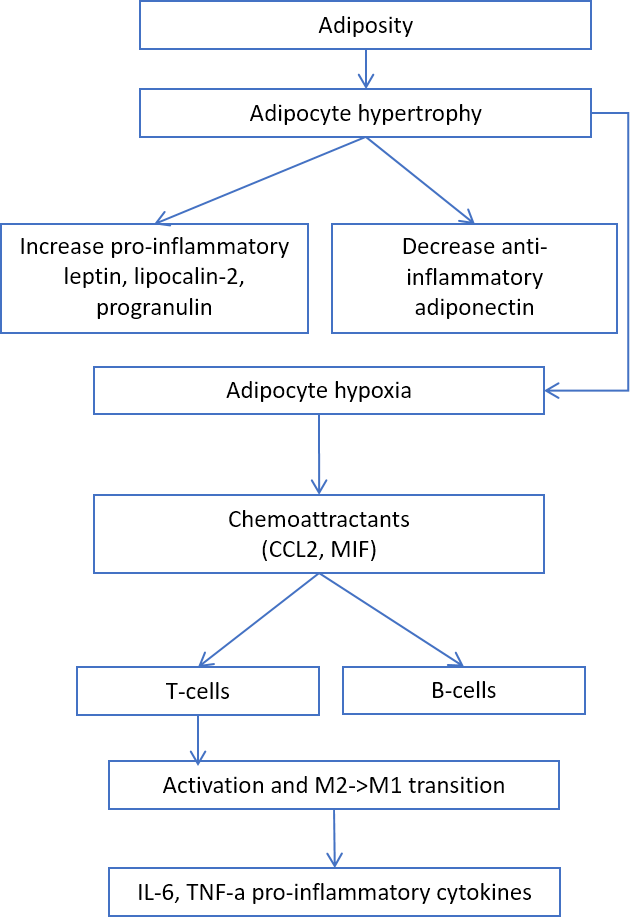
The role of adiposity in inflammation.

**Figure 5.**
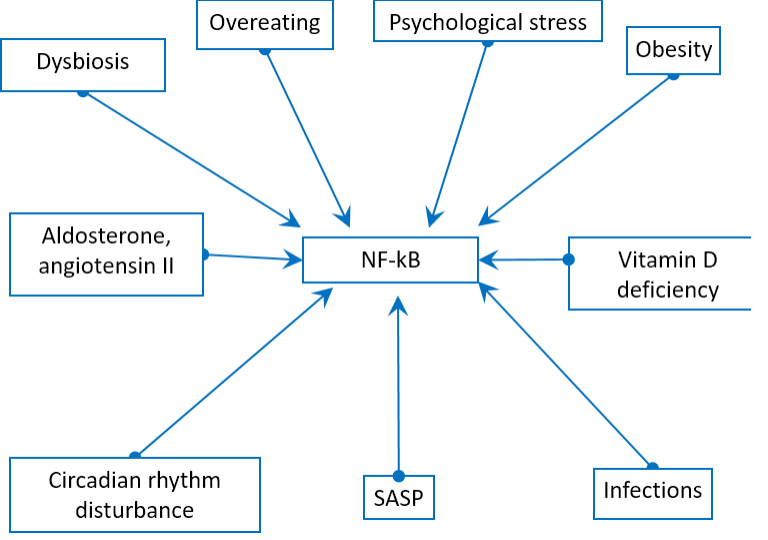
Physiological stress factors can induce NF-kB pathway.

Immune parameters associated with survival may vary in diverse populations of different ages. Therefore, we have to focus on the changes considered to be the hallmarks of immunosenescence, based on the literature data. The hallmarks of immunosenescence include: (i) a reduced ability to respond to new antigens; (ii) the accumulation of memory T cells; (iii) a lingering level of low-grade inflammation termed “inflamm-aging.” Mechanistically, immunosenescence is only partially explained by organismal and cellular senescence. Therefore, these hallmarks of immunosenescence would be markedly affected by the history of individual exposure to pathogens. In fact, several factors, such as genetics, nutrition, exercise, previous exposure to microorganisms, biological and cultural sex, and human cytomegalovirus (HCMV) status can influence immunosenescence [56].

Concerning sex/gender, in Western countries, women live 5-6 years more than men do. Furthermore, 85% of over 100 years old are women. It is debated whether women live longer than men for reasons of gender or sex, e.g., for cultural or biological differences. However, females live longer than males in other animal species. There is sexual dimorphism in the immune response, i.e. females are more resistant to infections, but they have a higher incidence of autoimmune diseases compared to males, yet their relevance for life span is negligible. However, age-related changes in various immunological parameters differ between men and women. Findings indicate that the slower rate of decline in immunological parameters in women than those in men is consistent with the fact that women live longer, than men do [57].

Concerning HCMV, virus status has a greater impact than age on the immune system because the virus contributes to shaping the immune profile and function during normal human aging. HCMV seropositivity is closely related to the reversal of the CD4/CD 8 T-cell ratio. In fact, persistent HCMV infection leads to chronic stimulation of CD8 T cells, which expand clonally showing an effector memory phenotype characterized by low CD28 expression. The absolute increase in memory T cells, called memory inflation, is observed only in older people infected by HCMV [58].

One of the pillars of adaptive immunity is the thymus. After an active period of creation and training of new T cells in childhood, at the time of puberty, the thymus undergoes involution, losing the stromal part and filling with fat [59], that can decrease T-cell repertoire to new antigens, including SARS-CoV-2. The involution is continuing during aging, because Wnt4 expression is down-regulated, while their Frizzled receptors and PPARgamma expression increases in the thymus [60]. On the contrary, peripheral T-cell numbers are maintained through the antigen-independent homeostatic proliferation of naive T cells that may lead to the emergence of dysfunctional memory-phenotype CD4+ T cell subpopulation (cell senescence-associated T cells, SA-T cells) [61]. SA-T cells secrete abundant pro-inflammatory factors such as osteopontin and chemokines, playing a direct role in SASP [62].

**Figure 6.**
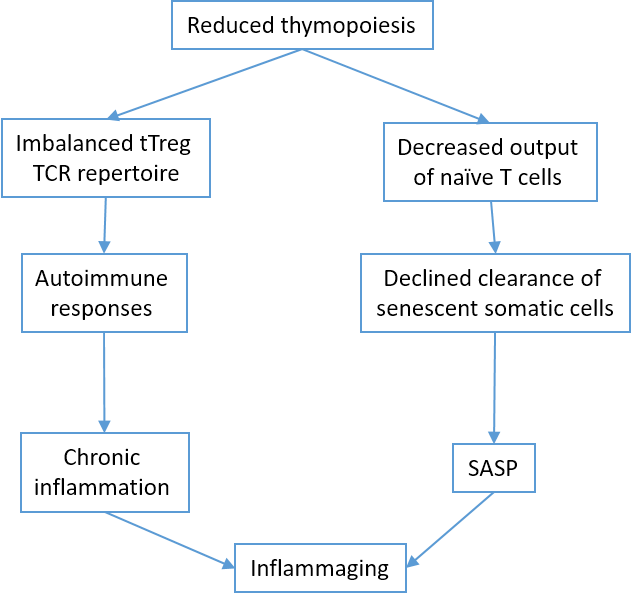
Thymus involution and inflamm-aging.

CD8+ cytotoxic T lymphocytes [63] and NK cells [64] clear cells infected by viruses (e.g. SARS-CoV-2). However, CD8+ themselves undergo senescence [65]. Immunosenescence could be the reason for the dysfunction of immune clearance of senescent cells [66]. In addition, senescent cells avoid immune clearance through HLA-E-mediated inhibition of NK and CD8 + T cells [67]. Thus, thymus involution is one of the mechanisms of inflamm-aging [68] (Fig. 6).

Senescence of bone marrow hematopoietic stem cells is affected by (HSC) niche [69] and intrinsic factors [70], extracellular matrix stiffness [71], systemic inflammation [72] or other systemic factors [73]. With age, HSCs reduce the homing and regenerative capacity and increase proinflammatory myeloid-biased differentiation [74].

T follicular helper (TFH) cells are presented in lymphoid organs and in peripheral blood and help B cells for the production of immunoglobulins. Dysfunctional TFH cells with aging play a role in cancer, autoimmune and cardiovascular diseases [75].

Recent studies revealed that long-lived mammalian species are characterized by the particularities in their immune system. Cancer and other age-related disease-resistant naked mole-rats lack canonical natural killer cells [76]. Many expanded gene families in the longest-living microbat *Myotis brandti* are involved in the immune response [77]. Bats showed a unique, age-related pattern of gene expression associated with DNA repair, autophagy, immunity and tumor suppression, which can lead to an increase in their health span [78]. They also express a reduced inflammation response after viral infection [79]. The evaluation of the bowhead whale genome revealed the potentially relevant changes in genes related to the immune response [80].

Human centenarians are a model for healthy aging. The longest living cohort of Italian centenarians has more favorable values of important immune parameters: naïve, activated/memory and effector/memory T cells [81]. Healthy centenarians presented with a distinct expression of proteins/pathways that reflect a healthy immune function, including less inflamm-aging and autoimmunity and increased B cell-mediated immune response [82].

**Figure 7.**
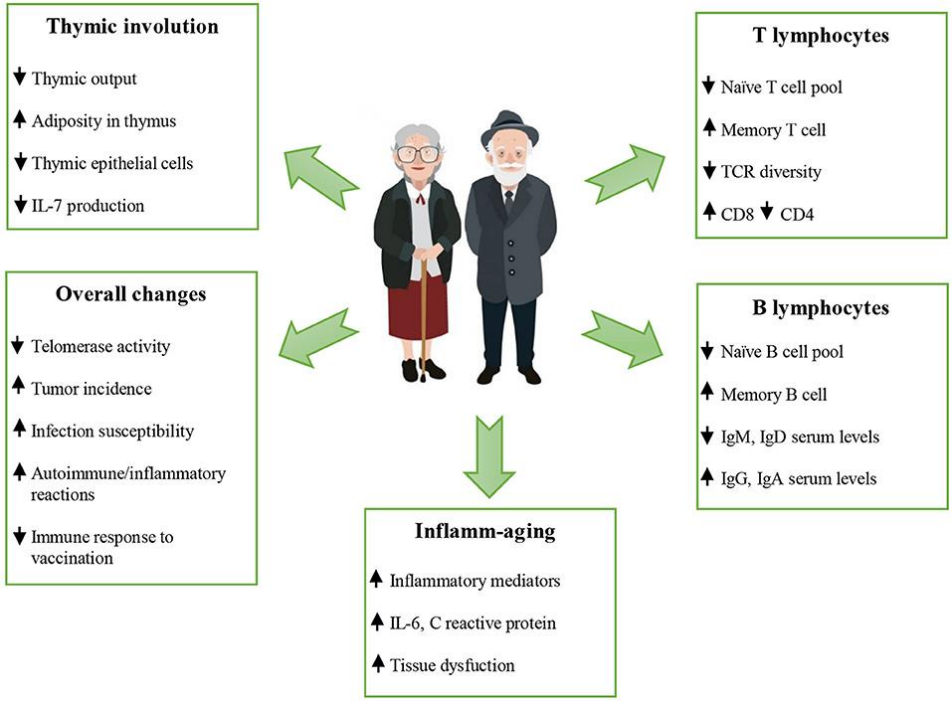
Changes occurring during aging (reproduced with permission from [56]).

Centenarians have been used as an optimal model for successful aging. However, this model shows several limitations, in particular the selection of appropriate controls. Thus, the interest has been centered on centenarian offspring, since it is well known that they are healthier than the remaining old people are. Accordingly, significant differences between old subjects and centenarian offspring, in most of the studied T and B subsets, show that centenarian offspring subsets present intermediate phenotyping between old and younger people. Therefore, centenarian offspring retain more youthful immunological parameters and the exhaustion of the immune system is less evident than in old people without centenarian parents [83]. Therefore, cell subset changes could represent a hallmark of successful or unsuccessful aging and could be used as a biomarker of human life span, potentially useful for the evaluation of immunosenescence treatment [83, 84].

Despite recent progress in understanding (Fig. 7), the harmonious theory of immunosenescence is still developing.

Based on the present level of knowledge, the geroprotective therapies targeting the mechanisms of immunosenescence are just emerging [85]. Their studies need to intensify, with a broader identification of potential clinically applicable interventions and biomarkers, and their extensive pre-clinical and clinical testing [85].

There are several hundreds of potential geroprotective interventions, that have been demonstrated on model organisms and collected in online databases Geroprotectors.Org [86] and DrugAge [87]. Not all of them meet the criteria of effective and safe treatment applicable for humans [88, 89].

According to the clinical studies conducted around the world, exercise, fasting, caloric restriction, resveratrol, metformin and NAD precursors are the interventions with the highest number of clinical trials that target aging [90]. For all of them, a geroprotective effect on immunity is shown. Epidemiological data indicate that regular physical activity reduces the incidence of infectious diseases in the elderly, including viral and bacterial infections, as well as non-infectious diseases associated with the immune system, such as cancer and chronic inflammatory diseases [91]. Cyclic fasting decelerated the immunosuppression caused by chemotherapy and reversed age-dependent myeloid-bias in mice [92]. Cycles of fasting reduce autoimmunity and activate the lymphocyte-dependent killing of cancer cells in humans [93]. The immunological status of rodents under calorie restriction is superior to the immunological status of the non-restricted animals, involving activation of the upstream signaling molecules and cytokine gene expression that are altered with age [94].

Arguably, there is no need to limit the entire diet. It is enough just to reduce the intake of certain nutrients to the necessary minimum. Protein restriction increased circulating interleukin-5 concentration in mice [95], that experimental overexpression *in vivo* significantly increases the number of eosinophils and B cells [96]. However, protein undernutrition is unfavorable for immune function in the elderly [97]. Methionine-deficient diet extends mouse lifespan and slows immune aging [98]. Branched-chain amino acid supplementation induced pro-inflammatory gene expression in visceral adipose tissue in mice [95]. On the contrary, treatment by other animo acids may decrease the aging-related loss of immune system function. Thus, alanine supplementation has stimulated the proliferation of immune cells [99].

Brian Kennedy et al. reviewed potential geroprotectors and paid special attention to rapamycin, senolytics, metformin, acarbose, spermidine, NAD+ enhancers and lithium [100]. Lithium presents a clear antiviral activity demonstrated at the preclinical level [101]. Lithium chloride confers protection against viral myocarditis via suppression of coxsackievirus B3 virus replication [102]. Lithium affects many aspects of immunity, including the activity of B- and T-cells, macrophages, interleukin-2 levels [103]. NAD precursors alleviate dysfunctional mitochondria in T cells [104]. Metformin enhances autophagy and normalizes mitochondrial function to alleviate aging-associated inflammation [105]. Acarbose benefits for immune function may be mediated by selective modulation of the gut microbiota [106]. After one year of treatment with acarbose or metformin, IL-6, TNF-α, IL-1β and ferritin levels of pro-inflammatory factors in type 2 diabetes patients were significantly decreased [107]. In accordance with a review [108], resveratrol can suppress the toll-like receptor and pro-inflammatory genes’ expression, associated with widespread health benefits for different autoimmune and chronic inflammatory diseases. Spermidine induces autophagy and improves the function of both the old mouse and old human B cells [109]. It has been proposed, that senolytics, i.e. drugs that selectively eliminate senescent cells that are the main source of pro-inflammatory cytokines with aging, may prove to alleviate immune dysfunction in older individuals [110]. However, this assumption requires experimental confirmation.

Rapamycin is a well-known potent immune-suppressive agent in xenotransplantation [111]. Rapamycin caused reversible thymus involution in mice [112]. Nonetheless, in a randomized control trial in an older human cohort, rapamycin increased a myeloid cell subset and T_REGS_ [113]. Network-based transcriptomic drug repurposing for novel coronavirus 2019-nCoV/SARS-CoV-2 revealed rapamycin along with melatonin and mercaptopurine as potential anti-HCoV drugs [114]. A large number of reports have documented a relationship between melatonin and the immune system [115].

Gaining this knowledge is urgently needed to enhance the quality of life and health span of the global aging population, to improve their resilience against both non-communicable and communicable diseases.
